# Photonanozyme–Kras–ribosome combination treatment of non-small cell lung cancer after COVID-19

**DOI:** 10.3389/fimmu.2024.1420463

**Published:** 2024-09-06

**Authors:** Qiaoyan Si, Mingjian Bai, Xiaolong Wang, Tianyu Wang, Yan Qin

**Affiliations:** ^1^ School of Chemistry and Biological Engineering, University of Science and Technology Beijing, Beijing, China; ^2^ School of Biomedical Engineering, Affiliated Cancer Hospital & Institute of Guangzhou Medical University, State Key Laboratory of Respiratory Disease, Guangzhou Medical University, Guangzhou, China; ^3^ Institute of Biophysics, Chinese Academy of Sciences, Beijing, China

**Keywords:** non-small cell lung cancer (NSCLC), photodynamic therapy (PDT), Kras mutants and targeted drugs, mammalian target of rapamycin (mTOR) signaling, ribosome, immunotherapy

## Abstract

With the outbreak of the coronavirus disease 2019 (COVID-19), reductions in T-cell function and exhaustion have been observed in patients post-infection of COVID-19. T cells are key mediators of anti-infection and antitumor, and their exhaustion increases the risk of compromised immune function and elevated susceptibility to cancer. Non-small cell lung cancer (NSCLC) is the most common subtype of lung cancer with high incidence and mortality. Although the survival rate after standard treatment such as surgical treatment and chemotherapy has improved, the therapeutic effect is still limited due to drug resistance, side effects, and recurrence. Recent advances in molecular biology and immunology enable the development of highly targeted therapy and immunotherapy for cancer, which has driven cancer therapies into individualized treatments and gradually entered clinicians’ views for treating NSCLC. Currently, with the development of photosensitizer materials, phototherapy has been gradually applied to the treatment of NSCLC. This review provides an overview of recent advancements and limitations in different treatment strategies for NSCLC under the background of COVID-19. We discuss the latest advances in phototherapy as a promising treatment method for NSCLC. After critically examining the successes, challenges, and prospects associated with these treatment modalities, their profound prospects were portrayed.

## 1 Introduction

Lung cancer is one of the most common and deadly cancer types in the world (1). According to the cell source, lung cancer can be divided into small cell lung cancer (SCLC) and non-small cell lung cancer (NSCLC), the latter accounting for approximately 85% of all lung cancer cases (2, 3). However, NSCLC can be further subdivided into lung adenocarcinoma, lung squamous cell carcinoma, and large cell carcinoma according to histological features (4). There are many risk factors for lung cancer, among which smoking (second-hand smoke) is the main risk factor for lung cancer (5). Approximately 80%–90% of lung cancer patients are related to active or passive smoking (6). Epidemiological studies indicate a significant age-related increase in lung cancer incidence (7). However, due to the pandemic of SARS-CoV-2 in 2020 (8), the elderly have become one of the most susceptible groups (9). Moreover, patients with lung cancer are at a higher risk of SARS-CoV-2 infection, and the mortality of lung cancer patients increased after COVID-19 infection (10–12). At the same time, those patients infected with COVID-19 exhibited a reduced total number of T cells and increased concentrations of IL-6 and IL-10, which are risk factors that aggravate the severity of COVID-19 in patients with lung cancer (13). Additionally, COVID-19 may alter the tumor microenvironment, promoting cancer cell proliferation and dormant cancer cell (DCC) reawakening. DCCs reawakened upon infection with SARS-CoV-2 can populate the premetastatic niche in the lungs and other organs, leading to tumor dissemination (14). Therefore, the treatment of cancer has become the focus of multidisciplinary research.

**Figure 1:**
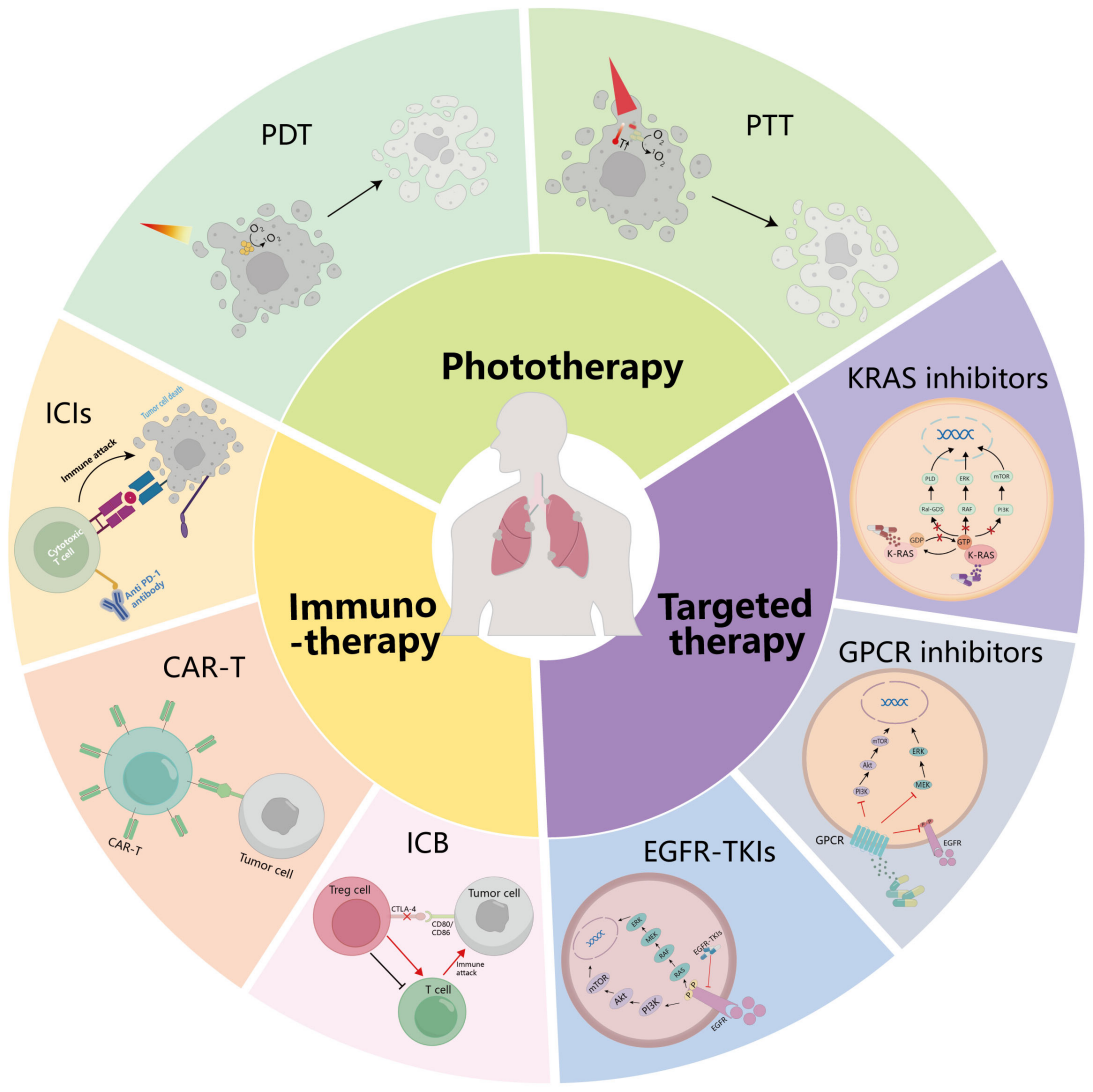
Summary of treatment modalities for NSCLC. The treatment methods of NSCLC are summarized: targeted therapy, immunotherapy, and phototherapy. GPCR, G protein-coupled receptor; EGFR, epidermal growth factor receptor; ICIs, immune checkpoint inhibitors; CAR-T, chimeric antigen receptor (CAR)-modified T cells; ICB, immune checkpoint blockade; PDT, photodynamic therapy; PTT, photothermal therapy.

There are more and more treatment options for lung cancer. In addition to traditional surgery (15), radiotherapy (16), and chemotherapy (17), targeted therapy (18), immunotherapy (19), phototherapy (20) (**Figure 1**), and other treatment methods have significant effects on lung cancer. In this paper, targeted therapy, immunotherapy, and phototherapy for NSCLC are summarized in detail, and treatments for NSCLC after the COVID-19 epidemic are prospected.

## 2 Targeted therapy for non-small cell lung cancer

Targeted therapy is a means of precise treatment at the molecular level, which is a common way to treat NSCLC. However, the impact of COVID-19 on the efficacy of targeted therapy remains unclear. Nevertheless, new targeted therapy is still the focus of attention in the treatment of NSCLC. In this section, we review three common modalities of targeted therapy for NSCLC, namely, EGFR-TKI, KRAS inhibitor therapy, and GPCR inhibitor therapy, and look forward to new research directions for these three treatment modalities.

### 2.1 EGFR-TKI

Epidermal growth factor receptor (EGFR) is a receptor for promoting mammalian epidermal growth factor, which mainly exists on the surface of human keratinocytes, epithelial cells, glial cells, fibroblasts, and other cell membranes (21). EGFR can be activated through pathways such as phosphatidylinositol-3-kinase (PI3K)/protein kinase B (AKT) pathway, rat sarcoma (RAS)/rapidly accelerated fibrosarcoma (RAF)/mitogen-activated protein kinase (MAPK) pathway, janus kinase (JAK)/signal transducer and activator of transcription (STAT) pathway, and phospholipase C-protein kinase C (PLC-PKC) pathway (22–25). These pathways transmit signals for cell proliferation and differentiation (26), which makes EGFR play a key role in tumor proliferation and survival. EGFR mutation is a key genetic alteration in non-small cell lung cancer (NSCLC), with mutation rate as high as 30% among NSCLC patients and approximately 16% among those with advanced lung adenocarcinoma (27). Most EGFR mutations in NSCLC patients occur in exons 18–21 of the receptor tyrosine kinase domain (28). The L858R mutation in exon 21 and deletion in exon 19 account for approximately 85% of EGFR mutations (29, 30). These mutations are recognized as oncogenic drivers of NSCLC and enhance the sensitivity to EGFR tyrosine kinase inhibitors (EGFR-TKIs).

The drugs for the treatment of NSCLC caused by EGFR mutations, epidermal growth factor receptor tyrosine kinase inhibitors (EGFR-TKIs), have gone through three generations of targeted drugs and are expected to usher in a fourth generation of targeted drugs (31) (**Table 1**). Among them, first-generation EGFR-TKI targeted drugs, such as gefitinib (32), erlotinib (33), and icotinib (34), targeting the typical mutations of EGFR, namely, the deletion mutation in exon 19 (delE746-A750) (35) and the point mutation in exon 21 (L858R) (36), have a good therapeutic effect. The clinical application of second-generation EGFR-TKIs, including afatinib (37) and dacomitinib (38), has been limited due to modest efficacy improvements and more serious side effects compared to first-generation drugs. These two generations of EGFR-TKIs inhibit the activation of EGFR by mimicking the structure of ATP and competitively interact with the binding site of the EGFR kinase domain, thereby inhibiting the occurrence of cancer. Concomitant drug use inevitably leads to the emergence of acquired drug resistance, and the T790M mutation is the most common, accounting for approximately 50%–60% of NSCLC cases (39, 40). The T790M mutation caused drug resistance due to the substitution of threonine (T) by methionine (M) at position 790 of EGFR, which hindered the binding of EGFR-TKI to EGFR. Meanwhile, the T790M mutation enhances the affinity between EGFR and ATP, which is also the reason for the decreased efficacy of EGFR-TKI (39, 41).

**Table 1:** Advantages and disadvantages of EGFR-TKIs targeted agents in the treatment of NSCLC induced by EGFR mutations.

Generation	Drug	Treatment	Advantage	Limitation
First	Gefitinib Erlotinib Icotinib	EGFR exon 19 deletion and exon 21 point mutation	Targeted therapies are effective	Susceptible to acquired drug resistance
Second	Afatinib Dacomitinib	As above	—	Dangerous side effects
Third	Osimertininb Almonertinib Furmonertinib	EGFR-sensitive mutation T790M resistance mutation	Resistance mutations can be treated	New drug-resistant mutations arise
No approved fourth-generation EGFR-TKIs on the market at present				
Fourth	EA1045	Overcoming osimertinib resistance	Binding to ATP is not dependent on Cys797, overcoming the generation of resistance mutations	Must be combined with cetuximab
	JBJ-04-125-02	EGFR exon 21 point mutation	combined with osimertinib and the osimertinib promotes its binding to cancer cells	Efficacy only for exon 21 mutation
	JBJ-09-063	EGFR exon 19 deletion	Inhibition of T790M drug-resistant mutations and unaffected by the C797S mutation	—
	TQB3804	T790M resistance mutation	Inhibition of drug resistance to the previous three generations of drugs	—
	BPI-361175	EGFR-C797S mutation	Effective in advanced stages of NSCLC	—

To solve the T790M acquired resistance mutation, the third-generation of EGFR-TKIs has been developed, including the famous osimertinib (42), almonertinib (43), and furmonertinib (44), which have been approved for marketing in recent years. At present, the third-generation EGFR-TKIs have been recommended by the authoritative lung cancer diagnosis and treatment guidelines of the United States and China as the first choice for the treatment of NSCLC with EGFR-sensitive mutation and T790M resistance mutation (45). Due to the widespread use of the drug, the development of resistance to the third-generation EGFR-TKI is also expected, and EGFR C797S mutation is the most common resistance mutation to osimertinib.

The fourth-generation EGFR-TKIs have been designed to treat patients who were resistant to first-generation EGFR-TKIs harboring the T790M mutation or to treat patients who were resistant to third-generation EGFR-TKIs harboring the C797S mutation. However, no fourth-generation EGFR-TKI has been approved for clinical application worldwide. Therefore, the treatment of EGFR-mutated non-small cell lung cancer should focus on solving EGFR-TKI resistance mutations.

### 2.2 KRAS inhibitors

**Figure 2:**
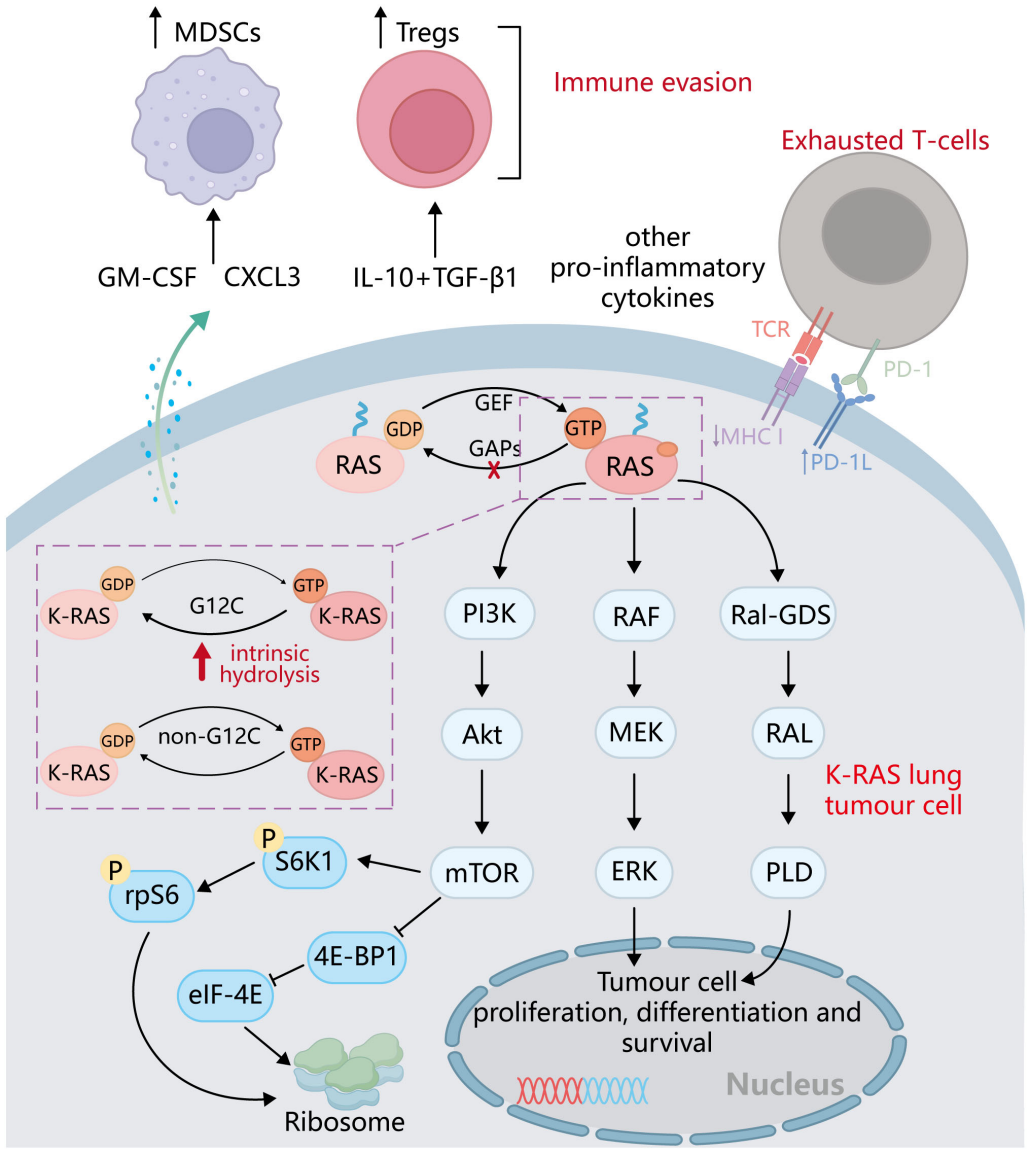
Major downstream pathways after RAS family activation: PI3K/Akt/mTOR, RAF/MEK/ERK, and RAL-GDS /RAL/PDL signaling pathways. After KRAS mutation is activated, it regulates the proliferation, differentiation, and survival of tumor cells through the above-mentioned major pathways and also affects the synthesis of ribosomes.

**Figure 3:**
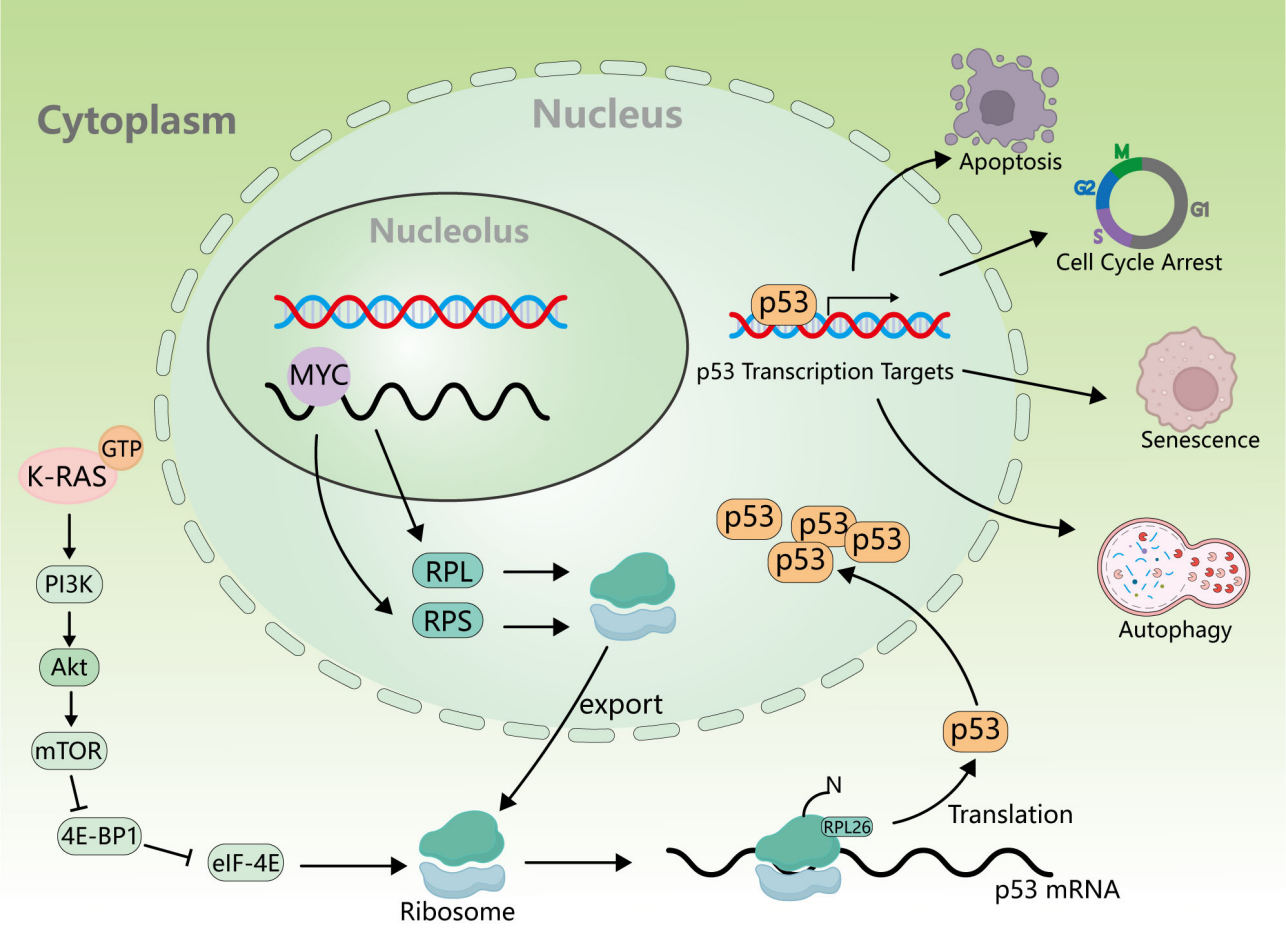
Relationship between KRAS activation and ribosomes and p53. KRAS activation affects ribosome synthesis through the PI3K/Akt/mTOR signaling pathway, and p53mRNA is translated on the ribosome to produce p53 protein, which further promotes apoptosis, cell cycle arrest, senescence, and autophagy.

The protein encoded by the KRAS gene is a small GTPase membrane-binding protein that plays a major role in cell growth regulation through signal transduction, and it belongs to the RAS superfamily of superproteins (46, 47). KRAS gene mutation accounts for 75% of the total number of RAS gene mutations (48), which is also a very common type of oncogene mutation. Specifically, KRAS gene mutation accounts for 20%–30% of the total number of NSCLCs, which is higher in lung adenocarcinoma and rare in lung squamous cell carcinoma (49, 50). In contrast, H-Ras mutations account for less than 1% (51, 52), and most H-Ras mutations occur on codon 61 (53). Due to the very low proportion of H-Ras mutations in NSCLC patients, there are relatively few research works on the clinical treatment of H-Ras mutations. Known targeted therapeutics for H-Ras mutation mainly focus on tipifarnib, a farnesyl transferase (FT) inhibitor, which is one of the most promising candidates (54, 55). However, no significant benefit was seen in people treated with tipifarnib, so tipifarnib is classified as a pan-Ras targeted drug (56). Therefore, the KRAS subtype is more concerned. KRAS G12C is the most common type of KRAS mutation (57). KRAS is a small GTPase, which cycles between inactivation (binding to GDP) and activation (binding to GTP) (58, 59). When KRAS is activated by binding to GTP, activated KRAS promotes RAF recruitment (60) and PI3K activation (61). Thus, it promotes cell proliferation, differentiation, and survival (62) (**Figure 2**). In addition, Ral-GDS has also been found to be an effector of KRAS, which is involved in vesicle trafficking and cytoskeleton composition (63). When KRAS mutation occurs, the ability of KRAS to hydrolyze GTP or interact with GDP will decrease, resulting in the continuous activation of KRAS and its downstream signaling factors and promoting tumorigenesis (64). At the same time, KRAS also influences ribosome production (65) through PI3K/Akt/mTOR, and the expression of ribosomal translated p53 protein is increased in cancer cells, contributing to both cancer generation and progression (66) (**Figure 3**).

For a long time, there was no good strategy to treat tumors caused by KRAS mutations. Until the introduction of inhibitors of the KRAS G12C mutation, a solution to tumors arising from KRAS mutation can be available. The FDA approved sotorasib (AMG510) for the first time for the treatment of tumors carrying KRAS G12C mutation (67). According to the results of clinical trials, AMG510 has a high disease control rate and good safety in NSCLC (68). Moreover, adagrasib (MRTX849) is a specifically optimized oral inhibitor of KRAS G12C mutant (69) by irreversibly and selectively binding to KRAS G12C in the inactive state, preventing it from sending cell growth signals and causing cancer cell death (70).

In addition to KRAS inhibitors targeting the G12C mutation, inhibitors targeting other mutations, such as G12D and G12V mutations, also have certain therapeutic effects on NSCLC caused by KRAS mutation (71, 72). However, these inhibitors are still in the stage of clinical trials, and there is still a long way to go before they can be approved for marketing. At the same time, the discovery of pan-KRAS inhibitors also provides another treatment for NSCLC (73) (**Table 2**). Because of the role of KRAS in ribosome formation, the combination of tumor-associated protein drugs and KRAS inhibitors could represent a novel approach to treating KRAS mutation-induced NSCLC.

**Table 2:** Different types of drugs targeting KRAS mutations and their research stages.

Drugs	Company	Properties	Status
AMG510 (sotorasib)	Amgen	G12C inhibitor	Approved for previously treated advanced-stage
MRTX849 (adagrasib)	Mirati Therapeutics	G12C inhibitor	Approved for previously treated advanced-stage
JNJ-74699157/ARS-3248	J&J and Wellspring Biosciences	G12C inhibitor	Phase I
JAB-21822	Jacobio Pharma	G12C inhibitor	Preclinical
D-1553	Inventis Bio	G12C inhibitor	Phase I
JDQ443	Novartis	G12C inhibitor	Phase I
RG6330	Roche	G12C inhibitor	Phase I
LY3537982	Eli Lily and Company	G12C inhibitor	Phase I
BI-1823911	BI	G12C inhibitor	Phase I
MK-1084	MSD	G12C inhibitor	Phase I
RM-018	Revolution Medicines	G12C inhibitor	Preclinical studies
RMC-6291	Revolution Medicines	G12C inhibitor	Preclinical studies
MRTX1133	Mirati Therapeutics	G12D inhibitor	Preclinical studies
JAB-22000	Jacobio Pharma	G12D inhibitor	Preclinical studies
RMC-9805 (RM-036)	Revolution Medicines	G12D inhibitor	Preclinical studies
mRNA-5671	Moderna Therapeutics	Cancer vaccine for G12C, G12D, G13D, G12V	Phase I, monotherapy and with PD-1 blocker pembrolizumab
BBP-454	Bridge BioPharma	Pan-KRAS inhibitor	Preclinical studies
RMC-6236	Revolution Medicines	Pan-KRAS inhibitor	Preclinical studies
BI-2865	BI	Pan-KRAS inhibitor	Preclinical studies

### 2.3 GPCR inhibitors

Seven transmembrane GPCRS constitute one of the largest families of extracellular signaling proteins in the human genome and regulate a large number of physiological and pathological processes. Therefore, they have also become one of the most attractive therapeutic targets for cancer treatment (74). GPCR can couple with G proteins to transmit a large number of extracellular signals produced by hormones, neurotransmitters, ions, photons, trend factors, and so on. Furthermore, GPCR can interact with heterotrimeric G proteins composed of α, β, and γ subunits, inducing the exchange of guanosine diphosphate (GDP) and guanosine triphosphate (GTP) at the Gα subunit. Then, Gα dissociates from Gβγ transduction signals to different downstream effectors, regulating the downstream signal transduction pathway (75). Thus, GPCRs serve as signaling hubs in cancer development by participating in cell proliferation, invasion, and migration and influencing immune cell-mediated functions (76, 77). Considerable evidence has shown that GPCR activation can promote the phosphorylation of its huge downstream corresponding factors, such as PI3k/Akt/mTOR (78) and PCK/RAF/MEK/ERK (79), thereby promoting the development of NSCLC—for example, Opsin 4/opiomelanocortin (OPN4) (79), a member of the Opsin family of GPCRs, is overexpressed in NSCLC patients and correlated with survival. The mechanism of OPN4 in the development of NSCLC has also been clearly elucidated, that is, OPN4 first binds to Gα11 to activate PCK, and then PCK phosphorylation activates its downstream signaling pathway BRAF/MEK/ERKs, which mediates NSCLC cell proliferation and migration. Moreover, Opsin4 inhibitor can effectively inhibit the proliferation and invasion of NSCLC cells.

In addition, much evidence suggests that GPCR ligand activation can also induce EGFR reactivation in various types of cancer cells, which is an important mechanism for regulating GPCR- and EGFR-mediated tumorigenesis (80, 81). Activated GPCR can upregulate EGFR ligand levels, thereby activating EGFR and its downstream signaling pathways (76, 82). In addition to their dependence on binding ligand activation, several adaptor proteins such as c-Src and β-arrestin were found to potentially participate in EGFR reactivation via GPCR (83, 84). Indeed GPCR interactions with EGFR contribute to the initiation and progression of colon, breast, ovary, prostate, and head and neck tumors (85). In NSCLC cells, GPCR transactivation of EGFR plays an important role in proliferation, migration, and drug resistance (86)—for example, protease-activated receptors (PARs), a subfamily of G protein-coupled receptors (GPCR), play important roles in hemostasis, thrombosis, embryonic development, wound healing, and inflammation (87). Among them, PAR2 activation is not only associated with arthritic pain, skin heat allergy, and paclitaxel-induced neuropathic pain (88, 89) but regulates a variety of downstream signaling pathways and cellular functions in a variety of cancer cells, including liver and renal cancer cells (90, 91). Meanwhile, many studies suggest that PAR2–EGFR interaction leads to COX2 expression, which promotes the proliferation of colon cancer cells (92, 93). However, PAR2 expression was significantly enhanced during the development of NSCLC and related to NSCLC patients’ survival (94). Meanwhile, in NSCLC, the activation of PAR2 leads to the reactivation of EGFR, which promotes the epithelial–mesenchymal transition (EMT) process of lung cancer cells through the ERK signaling pathway and then promotes the proliferation and migration of NSCLC cells (95). The β-blocker is an important regulator of PAR2-mediated ERK signaling activation following EGFR trans-activation. Therefore, PAR2 can promote the proliferation and migration of NSCLC through the β-arrestin-EGFR-ERK signaling pathway. At the same time, activation of ERK signaling and dysregulation of EMT are both considered to be the molecular mechanisms of resistance to osimertinib (94). Moreover, PAR2 plays an important role in the regulation of drug resistance in NSCLC by mediating the ERK signaling pathway. The use of the PAR2 inhibitor P2pal-18S can significantly attenuate the EMT process of NSCLC cells, reduce PD-L1 expression, and regulate cell viability, cell migration, and apoptosis. The simultaneous use of PAR2 inhibitors and NSCLC drugs, such as osimertinib and gefitinib, has demonstrated promising therapeutic effects on NSCLC even in cases of drug resistance (96). Therefore, the development of highly selective GPCR inhibitors has become a new frontier in targeted therapy NSCLC.

Certainly, targeted therapy also faces many challenges, especially drug resistance. In addition to the major challenge of drug resistance, other issues are summarized in **Table 3**. Firstly, because targeted therapy works by inhibiting a specific biomarker that is necessary for cancer progression, its effectiveness is limited to patients whose tumors express that biomarker (97). Secondly, even though targeted therapies can be effective for cancer therapy, unpredictable side effects are always inevitable. Common adverse events associated with targeted therapies include rash, diarrhea, hypertension, hypothyroidism, proteinuria, depigmentation, and hepatotoxicity (98). These side effects have both physical and mental impact on the treated patients. These problems continue to inspire researchers to innovate research to explore new ways of targeted therapy aimed at overcoming these existing limitations.

**Table 3:** Advantages and disadvantages of the three described treatment modalities for NSCLC (targeted therapy, immunotherapy, and phototherapy).

Treatment	Advantage	Disadvantage
Targeted therapy	Precise action on tumor cells, highly targeted	The small scope of application, susceptibility to resistance
Immunotherapy	Long-lasting therapeutic effect, minimal side effects	Long treatment time, inconsistent response during treatment
Phototherapy	Minimally invasive, convenient, remarkable therapeutic effect	Poor light penetration limits treatment, systemic photosensitivity, the lack of heat confinement

## 3 Immunotherapy for non-small cell lung cancer

Immunotherapy has revolutionized the treatment landscape for NSCLC, particularly in advanced or metastatic stages. It has significantly improved the survival rates and quality of life for many patients. Currently, immunotherapy options for NSCLC mainly include the following approaches: immune checkpoint inhibitors (ICIs), immune checkpoint blockade (ICB), and adoptive cell therapy (99).

### 3.1 ICI/ICB

**Figure 4:**
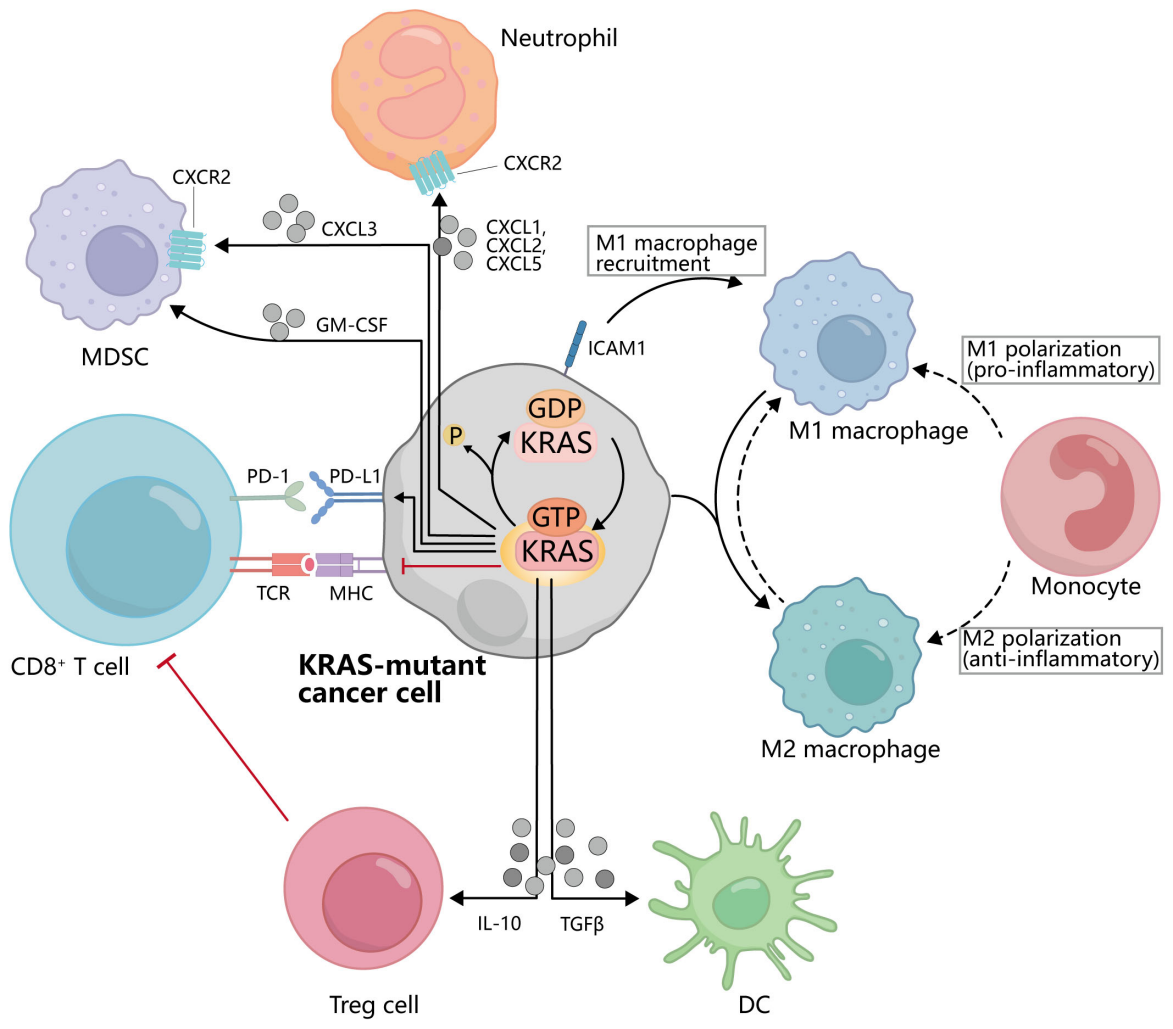
Interactions between KRAS-mutated cancer cells and immune cells. KRAS activation inhibits the binding of MHC on the cancer cell to TCR on CD8^+^T cells and promotes the binding of PD-1 to PD-L1. KRAS mutant cancer cells can secrete IL-10, TGFβ, GM-CSF, CXCL3, CXCL1, etc., to affect the activation and recruitment of Treg cells, DC, MDSC, and other cells. Meanwhile, the activation of KRAS also promoted the polarization of macrophages from M1 to M2.

With the rapid development of tumor immunotherapy, there are a variety of immunotherapy options for the first-line treatment of NSCLC, among which ICIs and ICB are the most promising (100). They were able to restore the ability of T cells to recognize and attack tumor cells by blocking immune checkpoint molecular interactions between tumor cells and T cells. Immune checkpoint molecules, such as programmed cell death protein 1 (PD-1)/programmed death ligand 1 (PD-L1) and cytotoxic T lymphocyte-associated protein 4 (CTLA-4), play a crucial role in regulating the immune response (101). They act as “brakes” to prevent excessive immune activation against healthy cells and tissues. However, cancer cells can exploit these immune checkpoints to evade the immune system and continue growing (102) (**Figure 4**). Therefore, ICI/ICB drugs block these immune checkpoint proteins, releasing the brakes on the immune system and allowing it to recognize and attack cancer cells more effectively (103).

The development of immunotherapies, particularly antibodies that inhibit the PD-1/PD-L1 pathways, has revolutionized the treatment of patients worldwide with advanced or metastatic NSCLC and improved their survival outcomes (104). Among ICIs, those targeting the PD-1/PD-L1 axis are the most promising, exerting potent anti-tumor effects by reversing T cell exhaustion and releasing anti-tumor immune responses (105, 106). PD-1 is a cell surface receptor expressed on activated T cells, B cells, and natural killer cells. Its ligands, PD-L1 and PD-L2, are primarily expressed in antigen-presenting cells (APCs) and tumor cells. The binding of PD-1 to PD-L1 or PD-L2 transmits an inhibitory signal to T cells, dampening their activation and effector functions (107). This signaling pathway plays a crucial role in maintaining immune homeostasis and preventing excessive immune responses. In the context of cancer, tumor cells often exploit the PD-1/PD-L1 pathway as an immune evasion mechanism. By expressing PD-L1, tumor cells can suppress and downregulate the immune response directed against them and escape destruction by cytotoxic T cells (108). The development of ICI/ICB targeting the PD-1/PD-L1 pathway has revolutionized cancer treatment, including NSCLC. For FDA-approved antibodies, two antibodies against PD-1 (nivolumab and pembrolizumab) and two antibodies against PD-L1 (atezolizumab and durvalumab) (109–112) have shown significant clinical benefits in NSCLC patients, with improved overall survival and progression-free survival compared to traditional chemotherapy in certain patient subgroups (113). These inhibitors have become standard treatment options, either monotherapy or in combination with other therapies, for advanced or metastatic NSCLC. However, it is worth noting that the response to PD-1/PD-L1 inhibitors varies among patients and tumor types (114). Factors such as tumor PD-L1 expression, tumor mutational burden, and the presence of other immune cells in the tumor microenvironment can influence the efficacy of PD-1/PD-L1 inhibitors (115).

Targeting the PD-1/PD-L1 pathway with immune checkpoint inhibitors has emerged as a promising therapeutic strategy for NSCLC and other cancers. These inhibitors have demonstrated significant clinical benefits by enhancing the immune response against tumor cells. Ongoing research aims to optimize the selection of patients to maximize the efficacy of PD-1/PD-L1 inhibitors and to develop strategies to overcome therapies’ resistance.

CTLA-4 is another immune checkpoint receptor that plays a critical role in regulating immune responses. It is expressed on the surface of T cells, where it acts as a negative regulator of T-cell activation. CTLA-4 binds to the B7 ligands (CD80 and CD86) expressed on APCs. This interaction leads to the inhibition of T-cell activation and the suppression of immune responses (116). By engaging CTLA-4, T cells become less responsive to antigen stimulation, leading to a dampening of the immune response and preventing excessive immune activation (117). Similar to the PD-1/PD-L1 pathway, cancer cells can exploit the CTLA-4 pathway to evade immune destruction by upregulating the expression of B7 ligands or expressing soluble CTLA-4, thus suppressing T cell activity and promoting tumor immune evasion (118). In NSCLC, although PD-1/PD-L1 inhibitors have shown remarkable efficacy in treatment, CTLA-4 is also considered a potential treatment target. Several clinical trials have investigated the efficacy of CTLA-4 inhibitors alone (such as ipilimumab) or in combination with PD-1/PD-L1 inhibitors in NSCLC patients. The results have shown significant clinical benefits in certain patient subgroups (NCT03527251). However, compared to other cancer types such as melanoma, the response to CTLA-4 inhibitors alone in NSCLC patients is not as prominent. This may be related to the differences in immune characteristics and antigen expression of NSCLC. Some studies have found that NSCLC patients with a high tumor mutational burden (TMB) may respond better to CTLA-4 inhibitors (NCT03527251 and NCT03913923). Tumors with high TMB have more nonsynonymous mutations, which may generate more neoantigens and trigger a stronger immune response (119). Therefore, high TMB may be an important indicator to predict the response to CTLA-4 inhibitors in NSCLC patients. Additionally, the therapeutic potential of CTLA-4 inhibitors is still under further investigation compared to PD-1/PD-L1 inhibitors. Currently, ongoing clinical trials are exploring the combination of CTLA-4 and PD-1/PD-L1 inhibitors to determine if it can produce better treatment outcomes in NSCLC patients (NCT03527251). Although the efficacy of CTLA-4 inhibitors alone in NSCLC patients is limited, combination therapy with PD-1/PD-L1 inhibitors may lead to improved treatment outcomes. By targeting both PD-1/PD-L1 and CTLA-4, it is possible to address different activation and inhibition mechanisms in T cells, further enhancing the anti-tumor immune response. Further research is still needed to identify which NSCLC patients are most suitable for receiving CTLA-4 inhibitor treatment and to make progress in defining treatment regimens and predicting patient responses.

Compared with conventional chemotherapy, ICI/ICB treatment significantly improved the survival outcomes in patients with advanced NSCLC. However, overall, there remains a subset of patients who do not respond to ICI/ICB therapy. Therefore, future clinical studies are needed to develop strategies to overcome immunotherapy resistance by identifying patients who are likely to respond to ICI/ICB therapy, explore new immune checkpoints and develop new immune checkpoint inhibitors or blockers for the treatment of NSCLC patients, and achieve a better understanding of the safety profile of ICI/ICB therapy to determine the proportion of patients who would benefit from expanding the ICI/ICB therapy (120, 121).

### 3.2 Adoptive cell therapy for non-small cell lung cancer

**Figure 5:**
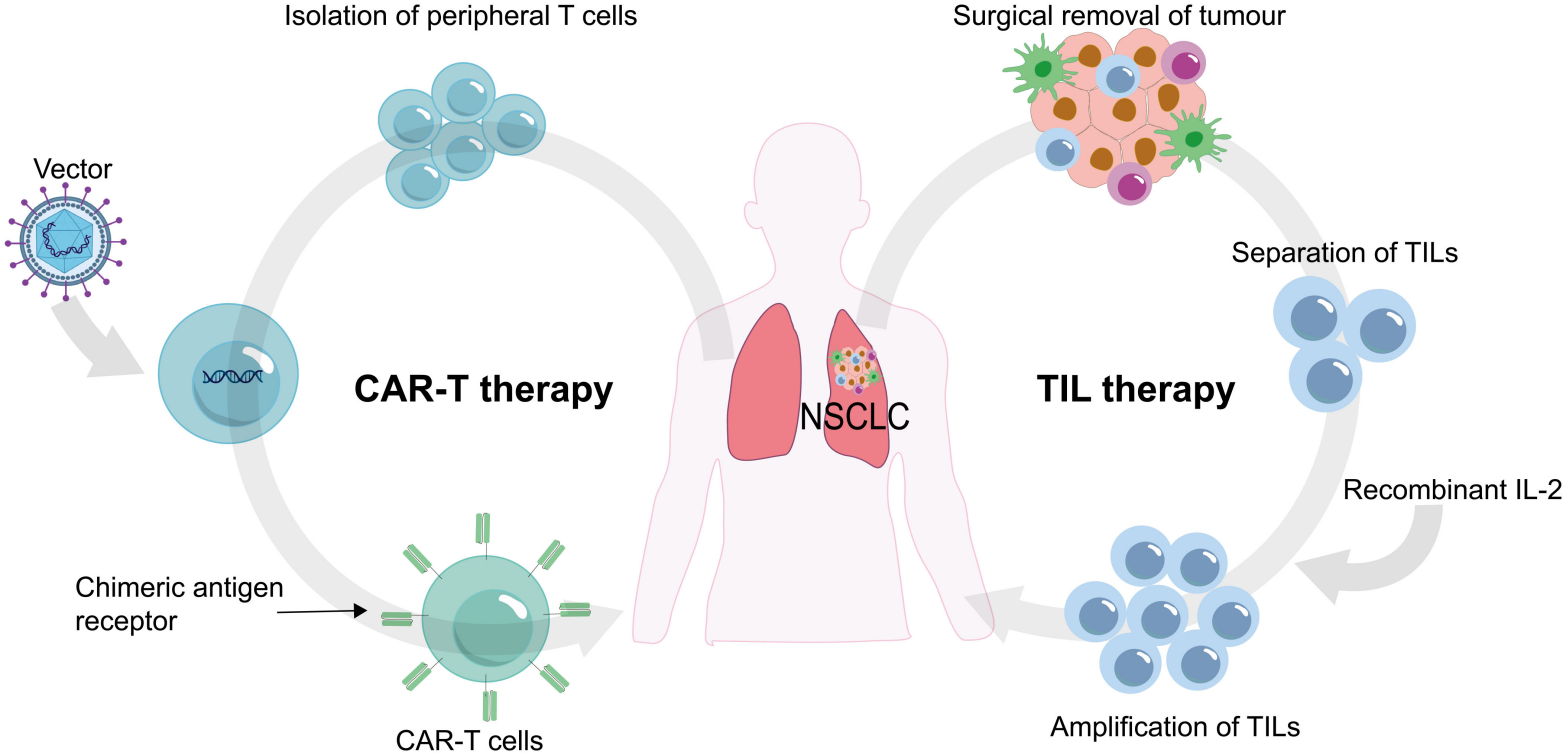
Chimeric antigen receptor (CAR) T cells and tumor-infiltrating lymphocytes (TILs) are procured from the patient's own body, subjected to laboratory processing, and subsequently reinfused back into the patient.

Adoptive cell therapy (ACT) involves utilizing immune cells (especially different types of T cells) that react to tumors. These cells are cultivated, grown, and genetically engineered outside the patient’s body before being re-administered as a therapy to identify and target cancer cells. The two most frequently employed forms of ACT are chimeric antigen receptor (CAR)-modified T cells (CAR T) therapy and tumor-infiltrating lymphocytes (TILs) therapy (122, 123) (**Figure 5**).

#### 3.2.1 CAR-T

CAR-T (chimeric antigen receptor T-cell immunotherapy) is an emerging tumor immunotherapy method (124–126). CAR-T involves genetically engineering T cells, which are immune cells in the human body, to specifically recognize and attack tumor cells (127). Researchers install a CAR (chimeric antigen receptor) onto the T cells, acting as a navigation system to identify and bind to specific antigens on the surface of tumor cells (128). Once modified, these CAR-T cells are expanded in large quantities and then infused back into the patient’s body to exert their anti-tumor effects. CAR-T therapy has shown significant efficacy in treating various types of cancers, particularly in hematologic malignancies, offering new hope and treatment options for patients. In addition, it has shown promising results in the treatment of various types of cancer, including NSCLC. In CAR-T cell therapy, T cells are extracted from a patient’s blood and modified in the laboratory to express CAR on their surface. The CAR is designed to specifically recognize a protein antigen that is expressed on the surface of cancer cells. Once the CAR-T cells are infused back into the patient, they can recognize and bind to the cancer cells, leading to their destruction (129, 130). CAR is an artificial fusion protein consisting of an extracellular antigen binding domain, an extracellular spacer sequence motif, a transmembrane (TM) domain, and an intracellular signaling domain. The TM structure and the hinge region fix the CAR to the cell membrane. The signal region is an intracellular T-cell activation complex composed of the signal module CD3ζ and many co-stimulatory molecules (131–133). They trigger antigen binding by regulating downstream signaling cascades activated by T cells. Co-stimulatory molecules include CD27, CD28, OX40 (CD134), and 4-1BB (CD137), inducing co-stimulatory molecules (CD278), and glucocorticoid-induced tumor necrosis factor (TNF) receptor-associated protein (GITR) (134). T cells modified with synthetic CAR vectors can recognize antigens specific to many human malignancies, including solid tumors (135–137), thus playing a therapeutic role in tumors. At present, CAR vectors have been upgraded to the fifth generation, differing mainly in their specific co-stimulatory molecular modifications.

The current fifth-generation CAR contains IL-2Rβ fragment modification. The IL-2Rβ fragment can induce the production of Janus kinase and transcriptional signal transducer and activator of transcription (STAT)-3/5 (138, 139) to enhance the efficacy of CAR-T therapy. The safety and efficacy of the fifth-generation CAR is being tested (140).

In solid tumors, CAR-T cell research mostly focuses on NSCLC (141). The most common antigens targeted by NSCLC include EGFR (142), mesothelin (MSLN) (143), carcinoembryonic antigen (CEA) (144), PD-L1 (145), human epidermal growth factor receptor 2 (HER2) (146), etc. CAR T cells bind to the antigens of NSCLC to treat NSCLC. In a study, the authors constructed CAR T cells targeting PD-L1 and evaluated their efficacy in the treatment of NSCLC. The results showed that PD-L1-CAR T cells effectively cleared PD-L1 NSCLC cells and xenograft tumors. Furthermore, the combination of PD-L1-CAR T cell injection with local radiotherapy enhanced the efficacy of PD-L1-CAR T cells against PD-L1 NSCLC cells and tumors (147).

CAR-T cell therapy offers several advantages over other forms of treatment. Firstly, CAR-T cells can specifically target cancer cells, sparing healthy cells from damage. Secondly, CAR-T cells can persist in the body and continue to attack cancer cells over a prolonged period. Lastly, CAR-T cell therapy can potentially induce long-term remission or even cure in some patients (122). However, there are still many obstacles in the application of CAR-T cell therapy in NSCLC, especially the specificity of antigens expressed by tumor cells. Therefore, improving the safety and efficacy of CAR-T cell therapy by optimizing CAR vectors is the direction of future development.

#### 3.2.2 TILs

TILs (tumor-infiltrating lymphocytes) are a subset of immune cells, primarily T cells, that migrate from the bloodstream and infiltrate the microenvironment of the tumor. These TILs can be found within the tumor mass itself as well as in the surrounding adjacent tissues (148). Once inside the tumor, TILs engage in intricate interactions with cancer cells and the surrounding tumor microenvironment. These interactions enable TILs to recognize specific antigens expressed by the cancer cells, subsequently triggering an immune response for combating the tumor (149). Compared to peripheral blood T cells, TILs often exhibit a higher concentration of T cell clones that target tumor antigens. This heightened specificity makes TILs a valuable resource for TILs therapy (150). This immunotherapy approach involves isolating TILs from the patient’s tumor, expanding them *ex vivo* with the assistance of recombinant IL-2 (rIL-2), and re-introducing amplified TILs into the patient to proliferate, recognize, and effectively eliminate the tumor cells (123).

A previous study examined the potential therapeutic effects of utilizing TILs as a post-operative intervention in patients diagnosed with stage II–III NSCLC. The study involved the surgical extraction of tissue samples from the primary lung lesions of these patients (151). After the isolation of lymphocytes, they were cultivated in a medium supplemented with rIL-2. The resulting TILs were then administered to patients who were stratified based on their disease stage. In addition, patients received daily subcutaneous injections of IL-2 until the maximum tolerable dose was reached. Notably, the TILs groups exhibited a more positive median survival outcome in comparison to the standard care treatment groups. These promising findings were further supported by a recent phase I trial characterized by a reduced tumor sample size. The trial demonstrated that TILs therapy, administered in doses ranging from 20 × 10^9^ to 20 × 10^10^ CD3^+^ cells and combined with IL-2, represented a viable treatment option with a manageable safety profile (152). It is important to note that the isolation and expansion of T cells from tumor tissue require advanced laboratory skills and a controlled environment. The preparation process is highly intricate and time-consuming, demanding a significant workforce. These reasons contribute to the high cost of treatment required for TILs therapy.

Immunotherapy can use the ability of the immune system to attack lung cancer cells and achieve therapeutic effects (153). Its advantages include a lower incidence of serious side effects than traditional therapy, longer efficacy than traditional therapy for specific patients, and the ability to be combined with traditional therapy. The disadvantages of immunotherapy include the need to test specific biomarkers to determine a patient’s suitability and the risk of triggering an autoimmune response. Immunotherapy is still in the early stage of development, and its efficacy needs to be confirmed by more studies (101, 154, 155) (**Table 3**).

## 4 Phototherapy for non-small cell lung cancer

Chemotherapy has been regarded as the first choice of standard lung cancer treatment, but resistance to chemotherapy is the biggest challenge in the treatment of lung cancer. The molecular mechanisms contributing to chemotherapy resistance include reduced drug activity and impaired drug uptake and delivery (156). These factors prevent the drug from reaching the desired concentration at the treatment site, thereby limiting its therapeutic efficacy (97). Chemotherapy drugs that do not reach the treatment site will throughout circulate the body and even appear in healthy organs and tissues, causing adverse reactions in patients with lung cancer and even endangering their lives (157, 158). To overcome the limitations of chemotherapy, researchers have discovered phototherapy. Phototherapy is a minimally invasive and effective treatment, a convenient method of ablating tumors with light irradiation in clinical practice (159). Phototherapy, including photodynamic therapy (PDT) and photothermal therapy (PTT), has attracted many researchers to study because of its being non-invasive, operability, and minimal dark toxicity in cancer treatment (160). Notably, PDT/PTT can potentially overcome chemotherapy resistance. Firstly, differently from chemotherapy, the effect of PDT/PTT can be dependent on the photophysical properties or photothermal characteristics of photosensitive drugs. The activity of PDT/PTT drugs cannot be decreased after multiple treatments (157, 161), and the cancer cells targeting of general photosensitizers (such as porphyrin derivatives) has been widely proven. Therefore, there is no decreased drug uptake for PDT (162). Most importantly, photodynamic therapy could significantly reduce the expression of ATP-binding cassette subfamily G member 2 (ABCG2), which is responsible for drug resistance in chemotherapy, resulting in enhanced chemotherapy effects and achieving reduced drug resistance (163). For cancer patients after COVID-19, due to the decrease in the number of T cells, the immune function is relatively weakened. However, it is unclear whether the efficacy of phototherapy for NSCLC will be diminished due to COVID-19. Therefore, this section mainly reviews photodynamic therapy and photothermal therapy for NSCLC in the hope that the impact of COVID-19 on human immunity can be considered when designing drugs for phototherapy in the future so as to maximize the efficacy of drugs.

### 4.1 PDT

**Figure 6:**
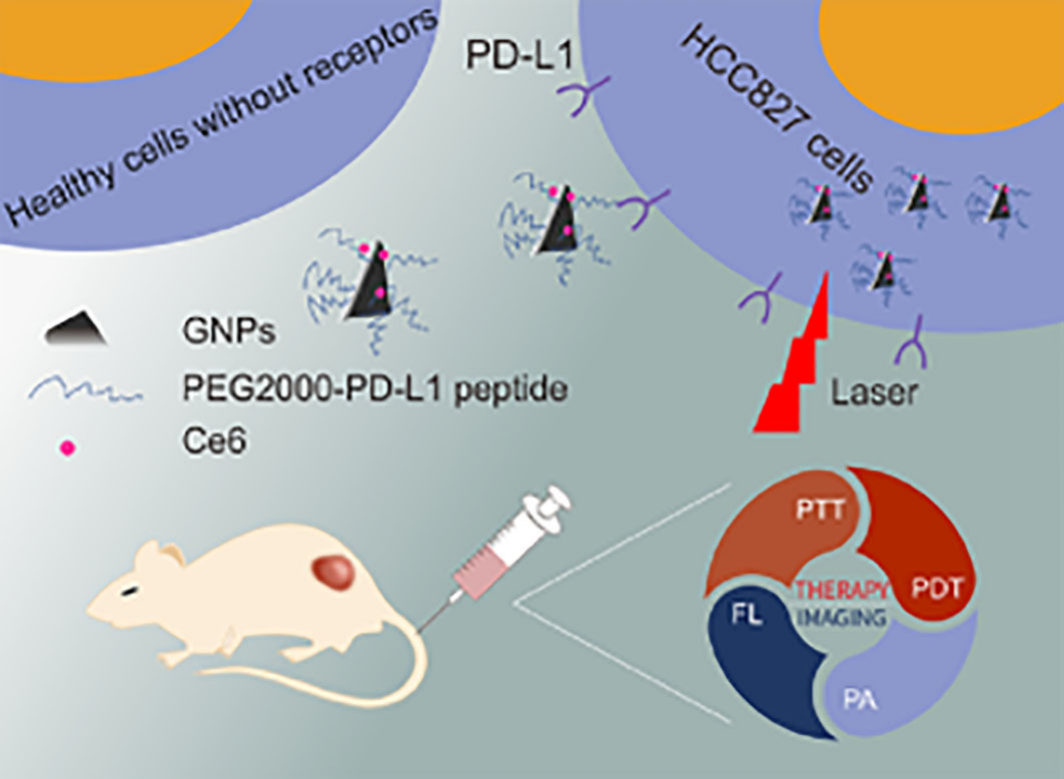
Schematic diagram of the synthesis process of GNPs@PEG/Ce6-P and its application in mice.

In PDT, photosensitizers undergo photoinduced electron transfer, usually in a physiological environment, to generate various ROS, including superoxide anion radical (·O_2_^-^), hydrogen peroxide (H_2_O_2_), and hydroxyl radical (·HO) (164, 165). There are three main cell death pathways in PDT, which are apoptosis, necrosis, and autophagy-related cell death pathways, of which apoptosis is the most commonly recognized (166, 167). At present, some small-molecule photosensitizers have been approved for clinical treatment, such as indocyanine green (ICG) (168), chloroe6 (Ce6) (169), porphyrin (170, 171), and other phototherapy drugs (172–174). However, its poor systemic circulation and limited targeting ability limit the output of its therapeutic effect. Therefore, more and more studies have been conducted to modify small-molecule photosensitizers with nano-agents to enhance their therapeutic effects and biological safety (175). A multifunctional nanoprobe GNPs@PEG/Ce6-PD-L1 peptide containing gold nanoprisms with chlorin e6 (GNPs@PEG/Ce6-P) (**Figure 6**) (176) was developed to exploit the targeting of PD-L1 for imaging-guided photothermal/photodynamic therapy. Moreover, the experiments *in vitro* and *in vivo* demonstrated that the nanoprobe GNPs@PEG/Ce6-P not only enabled real-time visualization via fluorescence and photoacoustic imaging but also served as a phototherapeutic agent for synergistic photodynamic therapy (PDT). In addition, the treatment of tumors derived from human lung cancer cells showed that the nanoprobe GNPs@PEG/Ce6-P could significantly inhibit tumor growth by PDT of GNPs and Ce6. It can be seen that the prepared novel nanoprobes have significant dual-mode imaging targeting ability and enhanced PDT effect on lung cancer, showing good prospects for nanomedicines (176).

**Figure 7:**
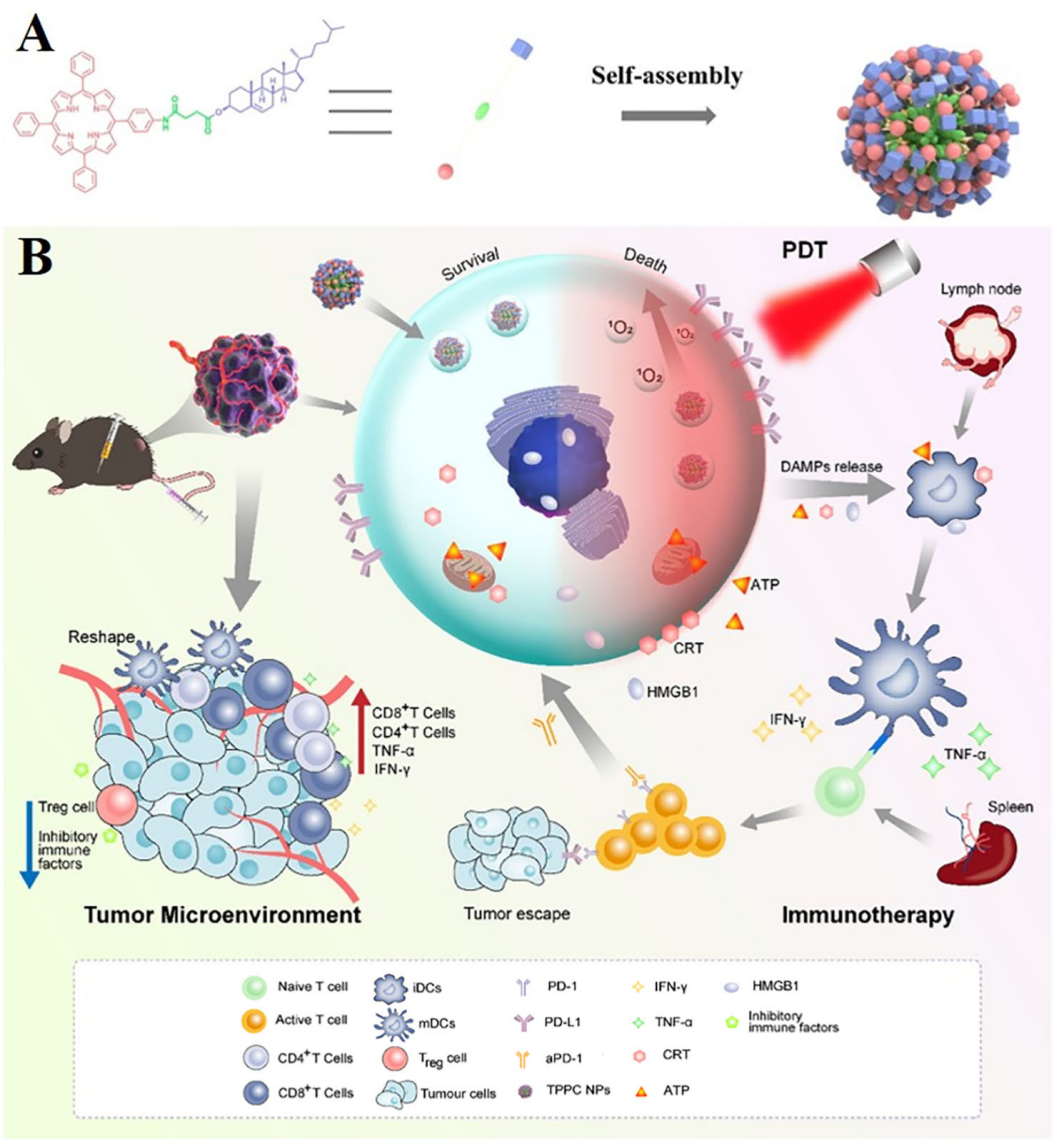
Porphyrin cholesterol conjugates (TPPC) for enhanced photodynamic immunotherapy toward non-small cell lung cancer. **(A)** Schematic illustration of preparation processes of TPPC NPs. **(B)** Schematic illustration of preparation and comparison of photodynamic immunotherapy of TPPC NPs.

Among various photodynamic therapy materials, nanomedicine based on cell membrane biomimetic strategy has received much attention due to its excellent ability to reduce the clearance of the reticuloendothelial system and enhance tumor targeting (177, 178)–for example, porphyrin cholesterol conjugate (TPPC) for synergistic photodynamic therapy (PDT) immunotherapy in lung cancer (**Figure 7A**) (179). Porphyrin derivatives have high generation efficiency of reactive oxygen species (ROS) and have been widely used as photosensitizers in clinical practice (169), while cholesterol is one of the main components of cell membranes. Porphyrin–cholesterol conjugates can be assembled into nanoparticles (NPS) in the absence of surfactants or amphiphilic polymers. Moreover, TPPC NP-mediated PDT can accumulate at the tumor site and induce immunogenic cell death, thereby stimulating and recruiting antigen-presenting cells to mature and activating T cells, making cancer cells more sensitive to ICI (**Figure 7B**) (179).

In addition to the modification of cell membrane components, hybrid membranes can also be used to modify nanomedicines. Except for the function of drug delivery to the body, hybrid membranes prepared by mixing and remodeling of different cell membranes can also be used for the homologous targeting of tumors through the homing effect of cancer cell membranes or immune escape of nanomedicines through macrophage membranes (180). PDT has many advantages, such as that mutations resistant to chemotherapy or radiotherapy do not limit PDT efficacy, and PDT treatments do not affect future therapy options. However, the limitations of PDT include the patient’s photosensitivity after treatment, bronchial tumor invasion of the pulmonary artery is not suitable for PDT treatment, and the treatment of thoracic malignant tumors depends on the location and size of the tumor (181).

### 4.2 PTT

PTT can reduce tumor burden by thermal ablation and has become an attractive option in cancer treatment (182). The high temperatures (>42°C) generated by photothermal agents by the local application of NIR light irradiation can lead to apoptosis of cancer cells by damaging cell membranes, disrupting the cytoskeleton, and inhibiting DNA synthesis (183–185). At the same time, PTT can trigger host immunity, revealing a broad prospect for combination with immunotherapy (186, 187). In addition, tumors are more sensitive to heat than normal tissues due to hypoxia and a weakly acidic microenvironment. Thus, this enhances the tumor selectivity of PTT (188).

The key to achieving tumor photoablation with PTT lies in the design of photothermal agents, which must meet the requirements of strong near-infrared light absorption, high photothermal conversion efficiency, good biocompatibility, and sufficient accumulation in the tumor. Currently, various types of inorganic and organic photothermal agents have been developed for their ability to perform PTT on tumors. Inorganic photothermal agents mainly include gold nanomaterials, such as Fe-based nanosphere (189), gold nanostars (AuNSs) (190, 191), and so on. Sulfide metal nanoparticles include CuS, MnS_2_, black phosphorus, etc. (192). In general, they are characterized by high photothermal conversion efficiency and good photothermal stability. Their photothermal properties can be tuned by changing their size, shape, or doping with other elements. Organic photothermal agents typically include small-molecule dyes such as indocyanine green (ICG) and IR780 as well as polydopamine (pD), polyaniline (PANI), and polypyrrole nanoparticles (193). These organic photothermal agents are generally degradable and biocompatible. In addition, photothermal agents are typically designed as nanoplatforms. These photothermal agents can be passively or actively targeted to tumors to enhance tumor accumulation owing to nanoscale size or surface modifications of targeting ligands such as antibodies, folate, peptides, and hyaluronic acid (194–196). In addition, they can simultaneously act as nanocarriers to load drugs, antigens, or adjuvants, showing the potential for combination therapy with other modalities (197, 198).

**Figure 8:**
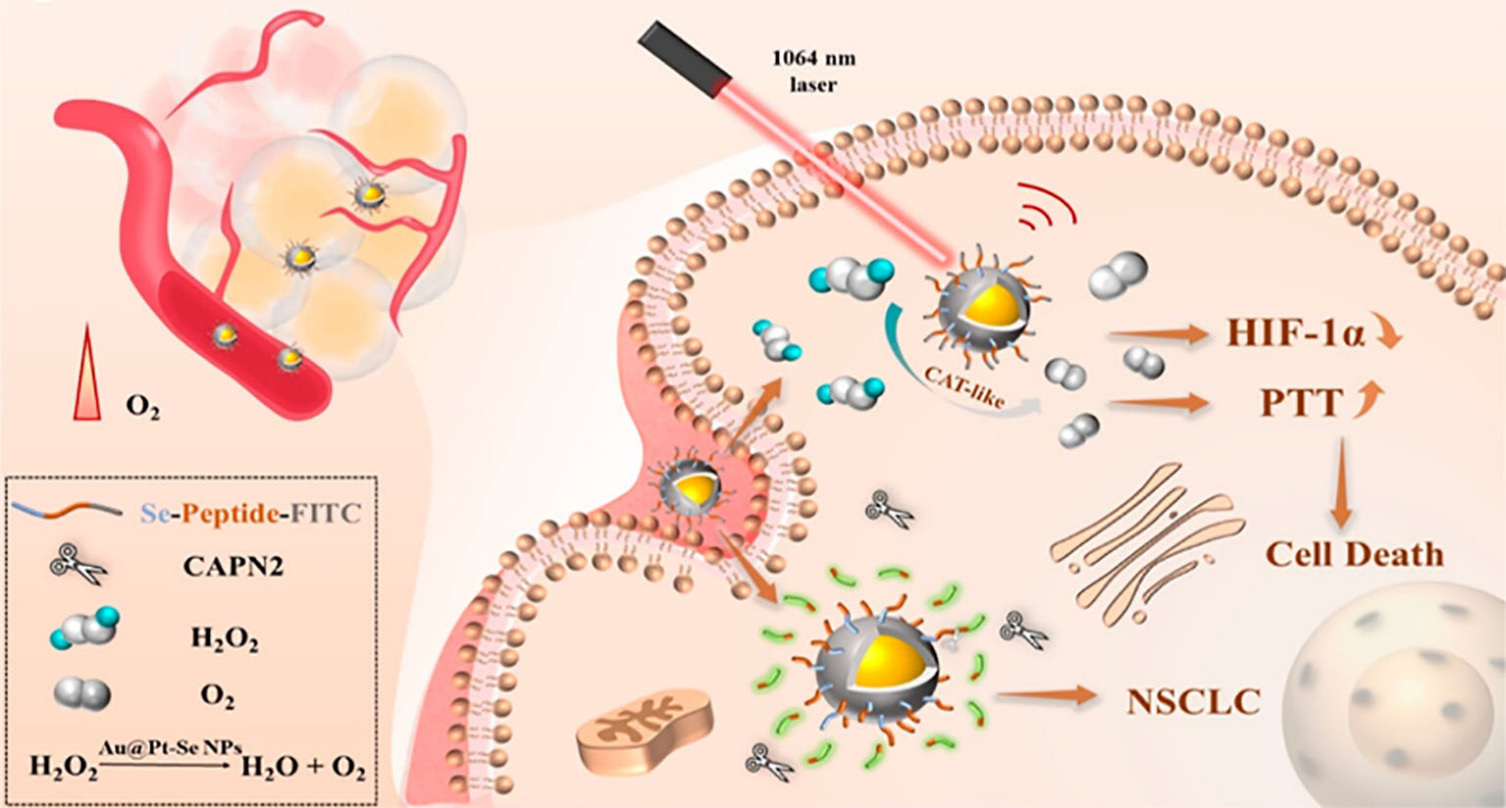
Schematic diagram of the effects of Au@Pt−Se NPs in NSCLS cells. Au@Pt-Se nanoparticles catalyze the generation of O_2_ from endogenous H_2_O_2_, alleviate the hypoxic environment, reduce the level of hypoxia-inducible factor-1α (HIF-1α), and improve the efficacy of PTT.

For example, core-shell Au@Pt−Se nanoprobes (Au@Pt−Se NPs) linked to peptide chains via Pt–Se bonds were designed and synthesized, which can be used to target NSCLC biomarker protein calcium-activated neutral protease 2 (CAPN2) and achieve photothermal therapy (PTT) enhancement (199). The rapidly grown tumors can exceed their vascular system, resulting in hypoxia at the tumor site (200). Although a variety of transcription factors are involved in the cellular response to hypoxia, hypoxia-inducible factor-1α (HIF-1α) is considered to be the most important transcription factor, which promotes tumor angiogenesis by upregulating pro-angiogenic genes such as vascular endothelial growth factor (VEGF) (201–203). Several studies have shown that blocking the activation of HIF-1α can significantly inhibit tumor angiogenesis and progression (204, 205). The platinum (Pt) shell has a catalase-like property and catalyzes the generation of oxygen from endogenous hydrogen peroxide within the tumor, thereby reducing the level of hypoxia-inducible factor-1α (HIF-1α) and alleviating the hypoxic environment at the tumor site (206–209). The Au@Pt−Se nanoparticles exhibit a strong absorption band, enabling PTT in the near-infrared II region (NIR II) (**Figure 8**). Moreover, according to the cell activity detection, the cell death rate of the NPs + H_2_O_2_ group and the laser group increased significantly, and the cell death rate was significantly higher than that of the other groups. This was consistent with the results of apoptosis rates measured by using flow cytometry (199).

Although PTT can rapidly reduce tumor volume, it is usually difficult to completely eradicate tumors with PTT alone, so PTT can be used in combination with other therapies to enhance the therapeutic effect on tumors.

**Figure 9:**
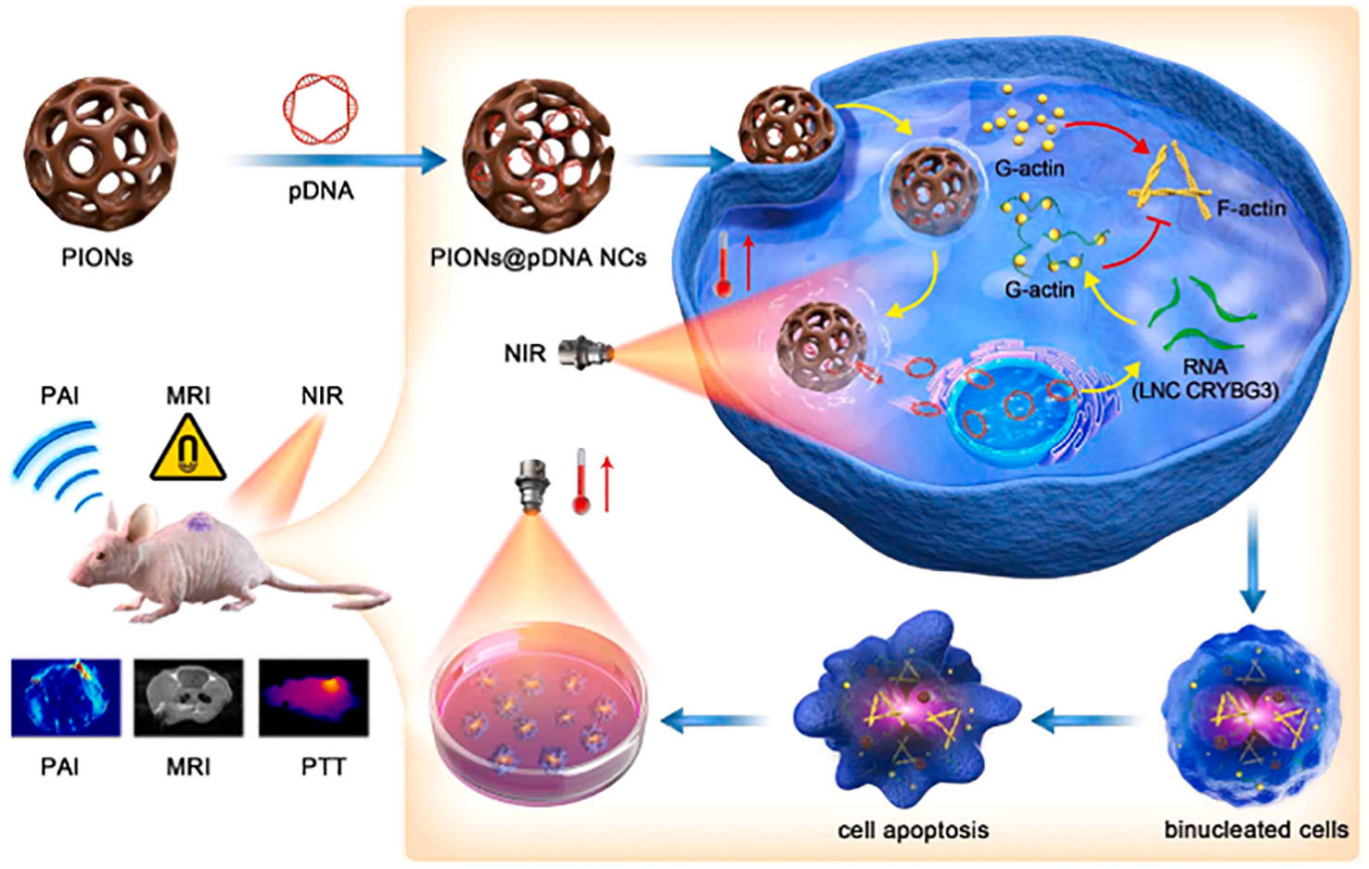
Schematic diagram of photothermal therapy using PIONs loaded pcDNA3.1-LNC CRYBG3 to synthesize PIONs nanocomplexes (PIONs@pDNA NCs) as photosensitizers.

For example, the integration of PTT and gene therapy (GT) on a single nanoplatform shows great potential in cancer therapy. Porous iron oxide nanoparticles (PIONs) are widely used as magnetic nanoparticles for drug delivery and can also be used as photothermal nanoparticles for photothermal therapy (210–213). However, the therapeutic effects of PION-mediated GT have not been investigated. Long non-coding RNA (lncRNA) CRYBG3 (LNC CRYBG3) is a lncRNA produced by heavy ion irradiation in lung cancer cells. It has been reported that it can directly bind to globular actin (G-actin), leading to cytoskeleton degradation and blocking cytodivision, indicating its strong gene therapy effect (214, 215). The potential of PION-mediated PTT and LNC crybg3-mediated GT to damage non-small cell lung cancer (NSCLC) cells *in vitro* and *in vivo* has been jointly applied (216). The combination therapy showed a high tumor-cell-killing effect and a better cure rate than PTT or GT alone. As a magnetic nanoagent, PIONs can be used for magnetic resonance imaging (MRI) and photoacoustic imaging (PAI) *in vitro* and *in vivo* (**Figure 9**). In addition, the tumor growth was almost completely inhibited in the PIONs@pDNA + NIR group in the animal experiment. These findings suggest that the combination of photothermal therapy (PTT) and gene therapy (GT) has a high potential for cancer treatment (216).

In addition to the above-mentioned specific examples, many various materials have been applied in PDT/PTT (**Table 4**).

**Table 4:** Various types of nanocarriers for PDT/PTT.

Materials	Applications	PDT/PTT	Reference
Liposomes/porphyrin assembly	*In vitro* and *in vivo* against SKOV3 cells	PDT	(224)
	*In vitro* and *in vivo* against A549 cells	PTT	(225)
Polymeric nanoparticles	*In vitro* against CaSki and HCT116 cells	PDT	(226)
	*In vitro* against TRAMP-C1 cells	PTT	(227)
Photosensitizer Micelles	*In vitro* against HepG2 cells	PDT	(228)
	*In vitro* and *in vivo* against 4T1 cells	PTT	(229)
Carbon-based nanomaterials	*In vitro* and *in vivo* against HT29 and LLC cells	PDT	(179, 230)
	*In vitro* and *in vivo* against 4T1 cells	PTT	(231)
Gold nanoclusters	*In vitro* against A549 cells	PDT	(232)
	*In vitro* and *in vivo* against HCC827 and HCT116 cells	PTT	(176, 233)
Photosensitizer/silica assembly	*in vitro* against MDA-MB-231 cells	PDT	(234)
	*In vitro* and *in vivo* against KB cells	PTT	(235)
Fe-MOF	*In vitro* and *in vivo* against 4T1 cells	PDT	(236)
	*In vitro* and *in vivo* against 4T1 cells	PTT	(237)
Protein-based nanoparticles	*In vitro* and *in vivo* against 4T1 cells	PDT	(238)
	*In vitro* and *in vivo* against A549 cells	PTT	(199, 216)

PDT/PTT therapy does not simply kill tumor cells by generating singlet oxygen or generating high temperatures. PDT/PPT can induce immunogenic cell death (ICD) by releasing calreticulin and exposing dead tumor cell debris, which triggers immunogenicity, leading to enhanced antigen presentation and T-cell activation to kill residual tumors (217–219). Moreover, the ROS (reactive oxygen species) generated during PDT treatments contributes to ICD and immune response (220).

Nevertheless, PDT/PTT alone, of course, is not the perfect way for the treatment of NSCLC. Firstly, the main limitation of any light therapy is the penetration limitation of the laser, which limits the role of PDT/PTT in treatment. PDT/PTT can only achieve a good therapeutic effect in the treatment of shallow epidermal tumors with rich blood vessels but cannot play a role in deep tumor therapy (221). Secondly, during PDT/PTT treatment, there are some difficulties in assessing the intratumoral drug concentration. Therefore, it may be complicated to evaluate the pharmacokinetics of some PDT/PTT drugs and subsequently interfere with the accurate clinical application of PDT/PTT (222). Finally, some safety problems exist in PDT/PTT treatment, such as general thermal constraints and lack of sensitivity of patients (223) (**Table 3**).

## 5 Discussion

After COVID-19, the treatments for NSCLC patients are encountering new changes and challenges. We have summarized and reviewed the main treatment methods for NSCLC and found that the treatment methods and therapeutic drugs for NSCLC are constantly updated, but drug resistance is still the biggest challenge in the treatment process. Therefore, the hot spot of NSCLC drug development in the future is still to break the bondage of drug resistance. Moreover, the therapeutic effect of single therapy for NSCLC patients is still limited, and combination therapy is the most favorable treatment for NSCLC patients. In addition, phototherapy, as a new treatment modality, has become the focus of research for the treatment of NSCLC. The biggest challenge for phototherapy to be used in the clinic in the future is whether the laser required for phototherapy can penetrate the skin to stimulate the photosensitizer to work. Therefore, exploring new phototherapy materials that can be applied in the clinic is the goal of researchers.
